# Spinophilin Is Indispensable for the α_2B_ Adrenergic Receptor-Elicited Hypertensive Response

**DOI:** 10.1371/journal.pone.0135030

**Published:** 2015-08-05

**Authors:** Pulin Che, Yunjia Chen, Roujian Lu, Ning Peng, Mary Gannon, J. Michael Wyss, Kai Jiao, Qin Wang

**Affiliations:** 1 Department of Cell, Developmental and Integrative Biology, University of Alabama at Birmingham, 1918 University Boulevard, Birmingham, AL 35294, United States of America; 2 the National Institute for Viral Disease Control and Prevention, China CDC, Beijing, China; 3 Department of Genetics, University of Alabama at Birmingham, 720 20^th^ Street South, Birmingham, AL 35294, United States of America; Loyola University Chicago, Stritch School of Medicine, UNITED STATES

## Abstract

The α_2_ adrenergic receptor (AR) subtypes are important for blood pressure control. When activated, the α_2A_ subtype elicits a hypotensive response whereas the α_2B_ subtype mediates a hypertensive effect that counteracts the hypotensive response by the α_2A_ subtype. We have previously shown that spinophilin attenuates the α_2A_AR-dependent hypotensive response; in spinophilin null mice, this response is highly potentiated. In this study, we demonstrate that spinophilin impedes arrestin-dependent phosphorylation and desensitization of the α_2B_AR subtype by competing against arrestin binding to this receptor subtype. The Del301-303 α_2B_AR, a human variation that shows impaired phosphorylation and desensitization and is linked to hypertension in certain populations, exhibits preferential interaction with spinophilin over arrestin. Furthermore, Del301-303 α_2B_AR-induced ERK signaling is quickly desensitized in cells without spinophilin expression, showing a profile similar to that induced by the wild type receptor in these cells. Together, these data suggest a critical role of spinophilin in sustaining α_2B_AR signaling. Consistent with this notion, our *in vivo* study reveals that the α_2B_AR-elicited hypertensive response is diminished in spinophilin deficient mice. In arrestin 3 deficient mice, where the receptor has a stronger binding to spinophilin, the same hypertensive response is enhanced. These data suggest that interaction with spinophilin is indispensable for the α_2B_AR to elicit the hypertensive response. This is opposite of the negative role of spinophilin in regulating α_2A_AR-mediated hypotensive response, suggesting that spinophilin regulation of these closely related receptor subtypes can result in distinct functional outcomes *in vivo*. Thus, spinophilin may represent a useful therapeutic target for treatment of hypertension.

## Introduction

The discovery of a plethora of G protein-coupled receptor (GPCR) interactions with non-G protein partners has led to the view that GPCRs do not function in isolation, but in complex protein networks which impact receptor trafficking, signaling and pharmacology. Among GPCR interacting proteins, G protein-coupled receptor kinases (GRKs) and arrestins are considered “universal regulators” and are the most extensively studied (e.g. recently reviewed in [1–6]). GRK-catalyzed phosphorylation, which leads to subsequent arrestin binding, represents a major mechanism for homologous desensitization of GPCRs. In addition to terminating G protein coupling, β-arrestins (arrestin 2 and 3) mediate receptor trafficking and scaffold cellular signaling cascades. Our previous studies identified spinophilin as an endogenous antagonist of arrestin, impeding multiple arrestin-mediated regulations of the receptor in cultured cells and *in vivo* [7]. Spinophilin is a ubiquitously expressed protein, and competes against arrestin for binding to the α_2A_ adrenergic receptor (AR) third intracellular (3i) loop [8]. Interestingly, multiple α_2A_AR-elicited central responses are dampened in arrestin 3 deficient mice, but these responses are potentiated in spinophilin deficient mice where arrestin functions are unimpeded [7, 9, 10]. This suggests that arrestin 3 promotes, whereas spinophilin attenuates, α_2A_AR-dependent processes *in vivo*.

Although closely related to the α_2A_AR, the α_2B_AR subtype exhibits distinct trafficking profiles [11–14] and induces separate physiological responses [15–17] from the α_2A_AR. In response to α_2_ adrenergic ligands, the α_2B_AR mediates the hypertensive effect [18], which counteracts the hypotensive response elicited by the α_2A_ subtype [19]. The α_2B_AR is also required for development of salt-induced hypertension [20–22]. In humans, a common polymorphism of the α_2B_AR gene, Del301-303, has been linked to early onset hypertension in a Swedish population [23, 24]. Unlike the α_2A_ subtype, the α_2B_AR 3i loop contains a highly acidic stretch of amino acids (aa294-309 [25]), which promotes GRK phosphorylation [25, 26]. Consistently, the Del301-303 α_2B_AR exhibits reduced phosphorylation and desensitization profiles [27]. The α_2B_AR interacts with both β-arrestins [13, 28] and spinophilin [29]. How spinophilin and β-arrestins regulate the α_2B_AR-mediated *in vivo* responses and how Del301-303 may affect the receptor’s interaction with these proteins remain to be investigated.

In the present study, we demonstrated that spinophilin impeded β arrestin-dependent α_2B_AR phosphorylation and desensitization by competing against arrestin binding to the receptor. Compared to the wild type (WT) α_2B_AR, the Del301-303 α_2B_AR exhibited diminished binding affinity to arrestin 3 but enhanced interaction with spinophilin. Moreover, ERK signaling induced by this polymorphic variant was prolonged. Intriguingly, Del301-303 α_2B_AR-induced ERK signaling was quickly desensitized in cells without spinophilin expression, showing a profile similar to that induced by the WT receptor in these cells. Together, these data suggest a critical role of spinophilin in sustaining α_2B_AR signaling. Furthermore, the α_2B_AR-elicited hypertensive response is diminished in spinophilin deficient mice, but the same response is enhanced in arrestin 3 deficient mice where the α_2B_AR has a stronger binding to spinophilin. These data strongly suggest that the interaction with spinophilin is indispensable for α_2B_AR to elicit the hypertensive response.

## Methods and Materials

### Reagents and drugs

Rat anti-HA rat monoclonal antibody (Roche); mouse HA.11 monoclonal antibody (Covance); rabbit anti-spinophilin antibody (Upstate); phospho- and total-p42/44 antibodies (Cell Signaling Technology); rabbit anti-GFP monoclonal antibody (Santa Cruz Biotechnology); mouse anti-myc antibody (Clontech); rabbit anti-GRK2 polyclonal antibody (Santa Cruz Biotechnology); anti-mouse IRDye 800CW and anti-rabbit IRDye 680RD (LI-COR) immobilized protein G-agarose (Pierce); arrestin 3 polyclonal antibodies (generously provided by Dr. Benovic, Thomas Jefferson University). All other chemicals were reagent-grade, and were purchased from Sigma-Aldrich or Fisher Chemicals.

### Animals

Spinophilin deficient (Sp^-/-^), arrestin 3 deficient (Arr3^-/-^) and their respective corresponding wild type (WT) mice in the same genetic background were obtained and maintained as described previously [7]. Mice were housed in the Association for Assessment and Accreditation of Laboratory Animal Care-accredited Animal Resources Program at the University of Alabama at Birmingham. Experimental procedures are in accordance with Animal Welfare Act and the 1989 amendments to this Act. All protocols were approved by University of Alabama Institutional Animal Care and Use Committee.

### Cells

HEK293 and CosM6 cells were originally from American Type Culture Collection (ATCC). Immortalized arrestin 2 and 3 double knockout (Arr2,3^-/-^) and the corresponding WT (Arr2,3^+/+^) mouse embryonic fibroblasts (MEFs) were generated [30] and generously provided by Dr. Lefkowitz’s laboratory. Sp^-/-^ and the corresponding Sp^+/+^ MEFs were generated previously as described in [31]. Cells were cultured in 5% CO_2_ at 37°C in DMEM (Invitrogen) supplemented with 10% fetal bovine serum (FBS) and 100 units/ml of penicillin and 10 μg/ml of streptomycin (Invitrogen). For mouse embryonic fibroblasts (MEFs), 2mM glutamine was added to the culture medium. Parental cell lines used in this study have no detectable endogenous expression of α_2_AR subtypes.

### Plasmid and primers

Constructions of plasmids pGFP-Arr3 [8], pCMV4-Myc-Sp [29, 32, 33], and pcDNA3-GRK2 [7] were described previously. The pGFP-Arr3-R170E construct expressing arrestin 3 with R to E mutation at R170 was generated by quickchange PCR mutagenesis using a primer pair, 5’-CTCAGGAGCAAACTGTACCTTCTCGATGATAAGCCGCACGGAGTT and 5’- AACTCCGTGCGGCTTATCATCGAGAAGGTACAGTTTGCTCCTGAG. pcDNA3.1- HA-α_2B_ expressing wild type human α_2B_AR with N-terminal 3xHA tag was purchased from UMR cDNA Resource Center (clone ID: AR0A2BTN00). pcDNA3.1-HA-Del301-303 expressing N-terminal tagged human α_2B_AR with deletion of amino acid 301–303 was generated by overlap PCR mutagenesis using two primer pairs, the 5’-CGGGGTACCACCATGTACCCATACGATGTT and 5’-ACACTCTTCCTCCTCCTCCTCCTCCTCTTCAGCTTCATCCT pair, and the 5’-GAGGATGAAGCTGAAGAGGAGGAGGAGGAGGAGGAAGAGTG and 5’-ATACCGCTCGAGTCACCAGGCCGTCTGGGTCC pair. All constructs were confirmed by sequencing.

### Transfection

Cells were transfected with indicated plasmid using Lipofectamine 2000 (invitrogen) according to manufacturer’s instruction. MEFs were transduced with a retroviral construct encoding HA-α_2B_AR as described previously [31, 34].

### Intact cell receptor phosphorylation

Intact cell phosphorylation was described previously [8]. Briefly, cells were incubated for 1 hr with [^32^p] orthophosphate (0.1mCi/ml) in phosphate-free, serum-free DMEM at 37°C. Following agonist treatment, cells were harvested in lysis buffer (1% Triton x-100, 0.05% SDS, 1mM EDTA, 1mM EGTA, 10mM NaF, 10mM sodium pyrophosphoate, and protease inhibitors). Detergent-soluble extracts were then subjected to immunoprecipitation assay with a rat anti-HA antibody.

### Co-immunoprecipitation

Co-immunoprecipitation was performed as described [8, 35]. In brief, cells were harvested in ice-cold lysis buffer (10 mM Tris HCl, pH 8.0, 0.3% Nonidet P-40, 10% glycerol, 5 mM EDTA, 5 mM EGTA supplemented with protease inhibitors). Soluble extracts were then subjected to immunoprecipitation assays using a rat anti HA antibody.

### ERK1/2 activation

Kinase activation for ERK1/2 was determined by measuring the level of phosphorylated kinase and normalizing the value to the total protein level of ERK1/2. Phospho- and total-ERK1/2 were detected and analyzed by LI-COR odyssey Fc dual-mode western blot system. Mouse anti-phospho-p44/42 MAPK (T202/Y204) (Cell signaling) and donkey anti-mouse IRDye 800CW (LI-COR) were used to detect activation of ERK1/2 by green fluorescence channel. Rabbit anti-p44/42 MAPK (Cell signaling) and goat anti-rabbit IRDye 680RD (LI-COR) were used to detect total ERK1/2 by red fluorescence channel.

### Measurement of cardiovascular responses

Measurement of cardiovascular responses was performed as described previously [9]. Mice were anesthetized with 100mg/kg ketamine and 10 mg/kg xylazine. Catheterized left femoral artery was used to measure arterial pressure while the right jugular vein was used for anesthetic administration. Arterial blood pressure was recorded with a pressure transducer (BIOPAC’s AcqKnowledge 3.8.2, BioPac, Goleta, CA) continually in conscious and free moving mice 24 hr after the surgery. Twenty minutes after the baseline measurement, 0.1mg/kg UK14,304 was administered through bolus injection into the right jugular vein.

### Data analysis

Data are expressed as mean ± SEM. Unpaired Student’s *t*-tests were performed to determine differences between two groups. All plots were generated using GraphPad Prism.

## Results

### Interactions of the α_2B_AR with spinophilin and β-arrestins are mutually exclusive

**Fig 1:**
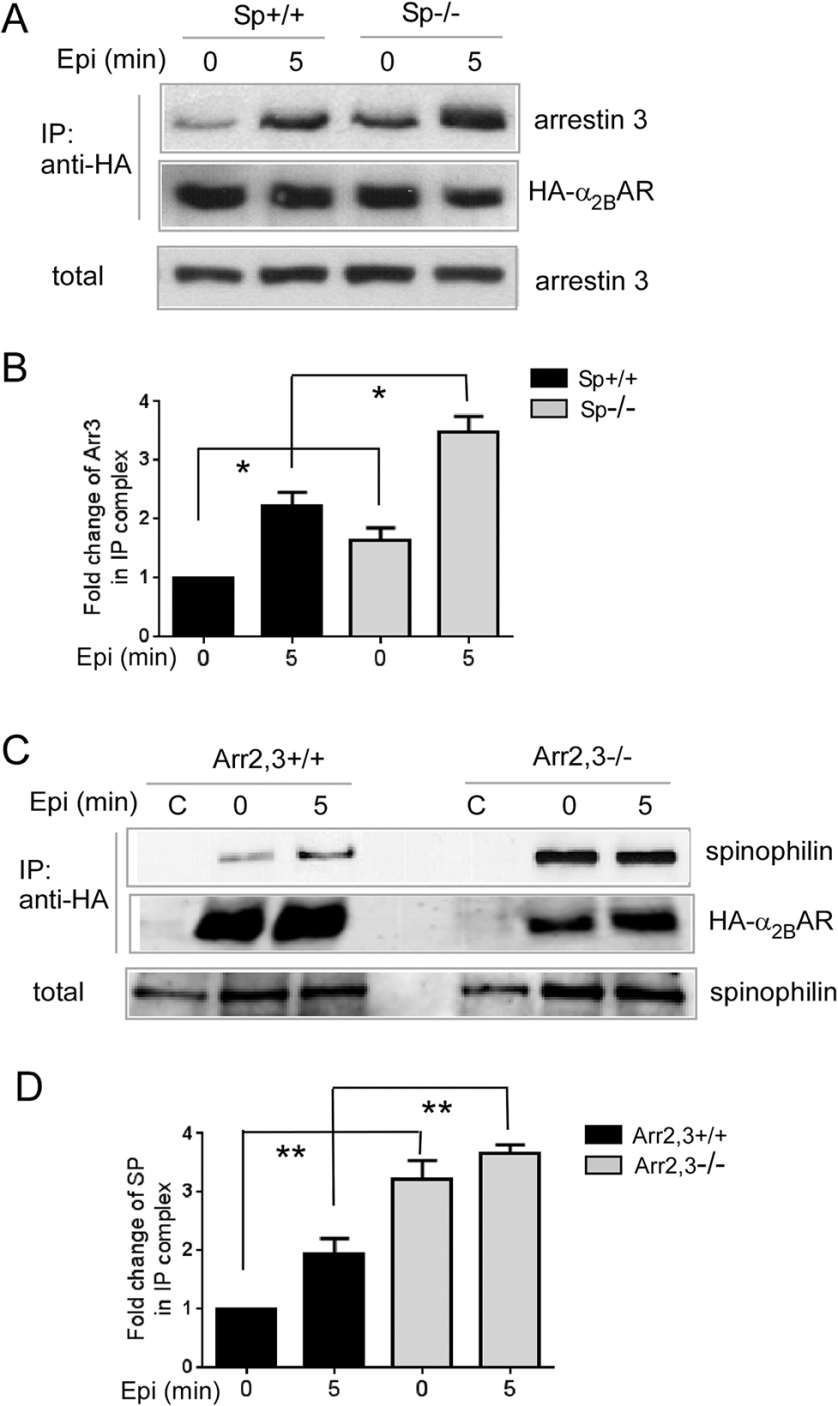
The endogenous arrestin and spinophilin competes for interaction with the α_2B_AR. (A) Interaction between α_2B_AR and the endogenous arrestin 3 was enhanced in Sp^-/-^ MEFs. Sp^-/-^ and corresponding WT (Sp^+/+^) MEFs expressing HA-α_2B_AR were stimulated with 100μM epinephrine (plus 1 μM propranolol to block βARs) for indicated time points. Cell lysates were subjected to IP assays using an HA antibody. (B) Quantitation of the fold change of arrestin 3 in the IP complex isolated from cells with or without stimulation. Data were mean ± SEM. n = 4 for each condition. *, *p*<0.05 by unpaired Student’s *t* test, Sp^-/-^ *vs*. Sp^+/+^. (C) Interaction between α_2B_AR and the endogenous spinophilin was enhanced in Arr2,3^-/-^ MEFs. Arr2,3^-/-^ and corresponding WT (Arr2,3^+/+^) MEFs expressing HA-α_2B_AR were stimulated with 100μM epinephrine (plus 1μM propranolol to block βARs). Lane C (control) refers to MEFs (Arr2,3^+/+^ or Arr2,3^-/-^) without HA-α_2B_AR overexpression. (D) Quantitation of the fold change of spinophilin in the IP complex isolated from cells with or without stimulation. Data were mean ± SEM. n = 4 for each condition. **, *p*<0.01, Arr2,3^-/-^ *vs*. Arr2,3^+/+^.

We first confirmed that β-arrestins and spinophilin compete for interaction with the α_2B_AR in cells. MEFs express endogenous arrestins and spinophilin, and we readily detected interactions of the α_2B_AR with endogenous arrestin 3 (Fig 1A, left) and spinophilin (Fig 1C, left), which were enhanced by epinephrine stimulation. In spinophilin deficient (Sp^-/-^) MEFs, the epinephrine-promoted interaction between the α_2B_AR and arrestin 3 was markedly increased, as compared to that in the corresponding Sp^+/+^ MEFs prepared from WT mice with the same genetic background (Fig 1A, right, and Fig 1B). Similarly, the association of the α_2B_AR with spinophilin in response to epinephrine treatment was significantly enhanced in MEFs with no β-arrestin expression (Arr2,3^-/-^), as compared to that in the corresponding Arr2,3^+/+^ MEFs (Fig 1C, right, and Fig 1D). We obtained similar results with other α_2_ agonists, including clonidine and UK14,304 (data not shown). Additionally, in the absence of arrestin, the basal interaction between α_2B_AR and spinophilin was also dramatically enhanced (Fig 1C and 1D). Together, these data demonstrate that interactions of the α_2B_AR with spinophilin and β-arrestins are mutually exclusive.

### Spinophilin attenuates α_2B_AR phosphorylation through competition against arrestin in cells

**Fig 2:**
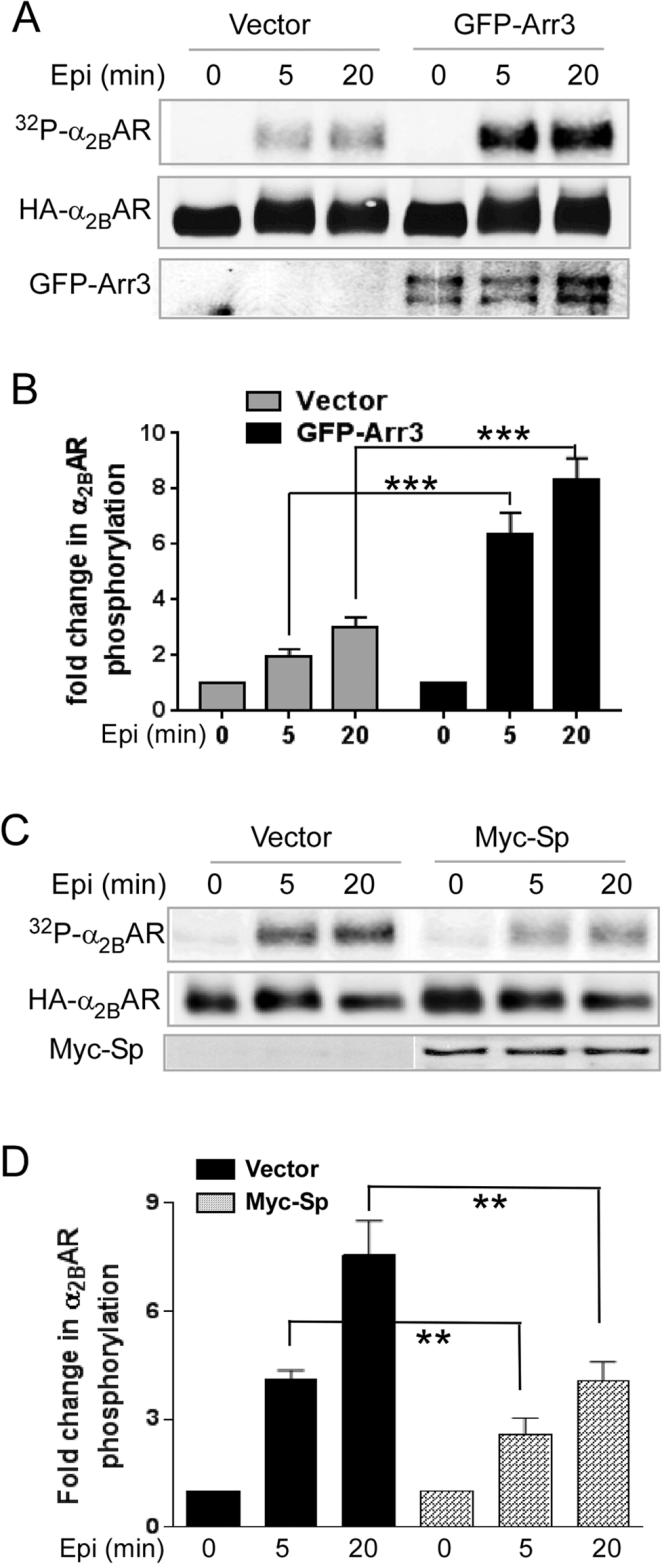
Arrestin 3 and spinophilin reciprocally regulate agonist-induced α_2B_AR phosphorylation. (A) CosM6 cells co-expressing HA-α_2B_AR together with GFP-tagged arrestin 3 (GFP-Arr3) or GFP alone (vector) were stimulated with 100μM epinephrine (plus 1μM propranolol to block βARs) for indicated time points. Overexpression of GFP-Arr3 increased the phosphorylation level of α_2B_AR following epinephrine stimulation. (B) Quantitation of agonist-induced fold change in α_2B_AR phosphorylation. Data were mean ± SEM. n = 5 for each condition. ***, *p*<0.001 by unpaired Student’s *t* test, GFP-Arr3 *vs*. vector control. (C) HEK293 cells co-expressing HA-α_2B_AR with or without Myc-spinophilin were stimulated. Overexpression of Myc-spinophilin (Myc-Sp) reduced the phosphorylation level of α_2B_AR following epinephrine stimulation. (D) Quantitation of agonist-induced fold change in α_2B_AR phosphorylation. Data were mean ± SEM. n = 3 for each condition. **, *p*<0.01, Myc-Sp *vs*. vector control.

We previously demonstrated that stable phosphorylation of the α_2B_AR requires β-arrestins [7]. Consistently, when arrestin 3 was overexpressed in CosM6 cells, which have a relatively low level of endogenous arrestins compared to other cell lines such as HEK cells and MEFs [7, 36, 37], the levels of α_2B_AR phosphorylation following epinephrine stimulation were dramatically enhanced compared to the control (Fig 2A and 2B). On the other hand, when spinophilin was overexpressed in HEK293 cells, epinephrine-induced α_2B_AR phosphorylation was markedly reduced compared to the control (Fig 2C and 2D). This result is consistent with the notion that spinophilin competes against β-arrestins for binding to the α_2B_AR and impedes the arrestin effect in promoting receptor phosphorylation.

**Fig 3:**
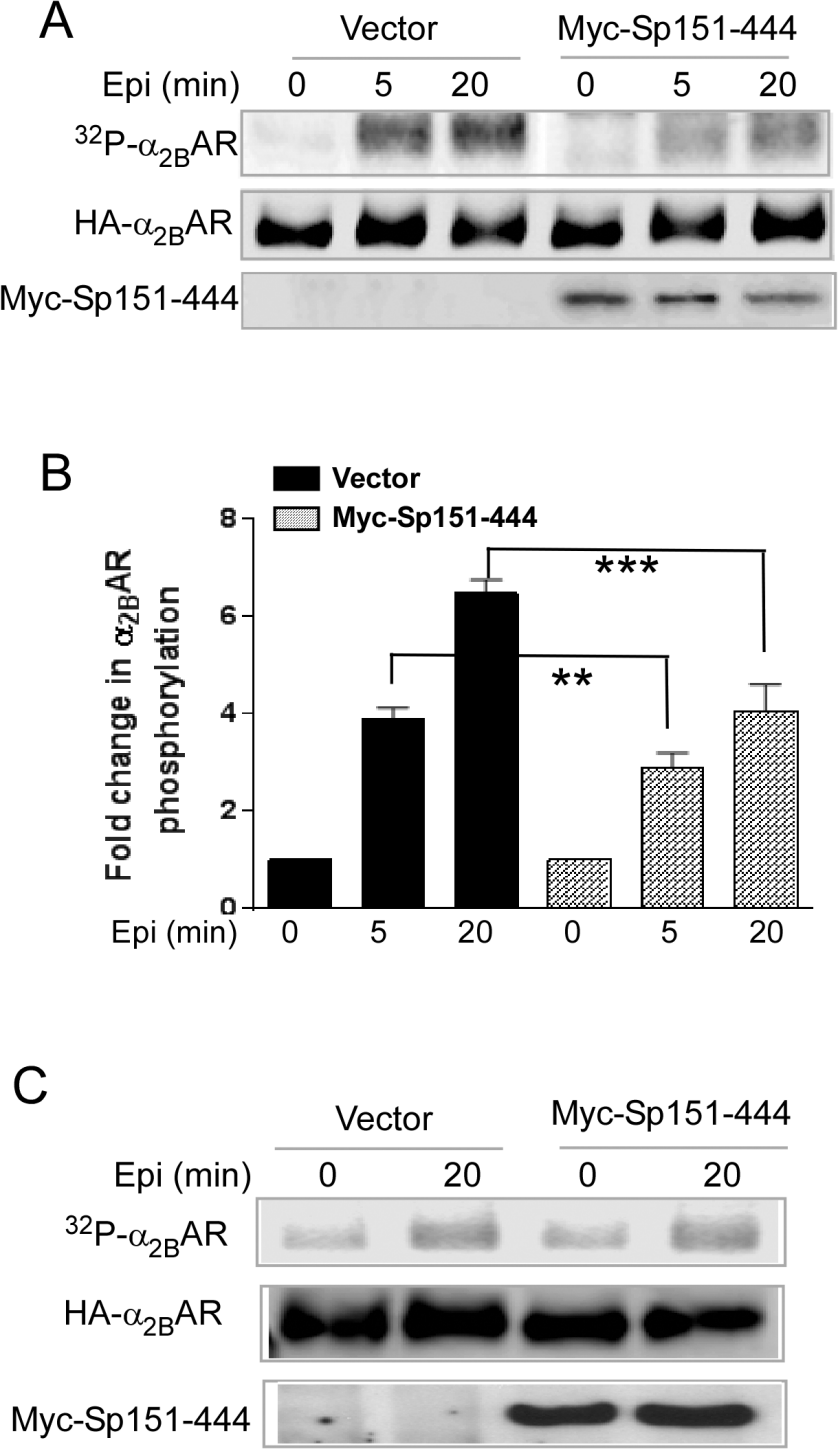
Overexpression of spinophilin aa151-444 sufficiently attenuates α_2B_AR phosphorylation. (A) HEK293 cells co-expressing HA-α_2B_AR t.ogether with or without Myc-Sp151-444 were stimulated with 100μM epinephrine (plus 1μM propranolol to block βARs) for indicated time points. (B) Quantitation of agonist-induced fold change in α_2B_AR phosphorylation. Data were mean ± SEM. n = 4 for each condition. **, *p*<0.01; ***, *p*<0.001 by unpaired Student *t* test, Myc-Sp151-444 *vs*. vector control. (C) Overexpression of Sp151-444 showed no effect on α_2B_AR phosphorylation in CosM6 cells, which have a low level of endogenous arrestin expression. CosM6 cells co-expressing HA-α_2B_AR together with or without Sp151-444 were stimulated. Representative blots from multiple independent experiments are shown.

**Fig 4:**
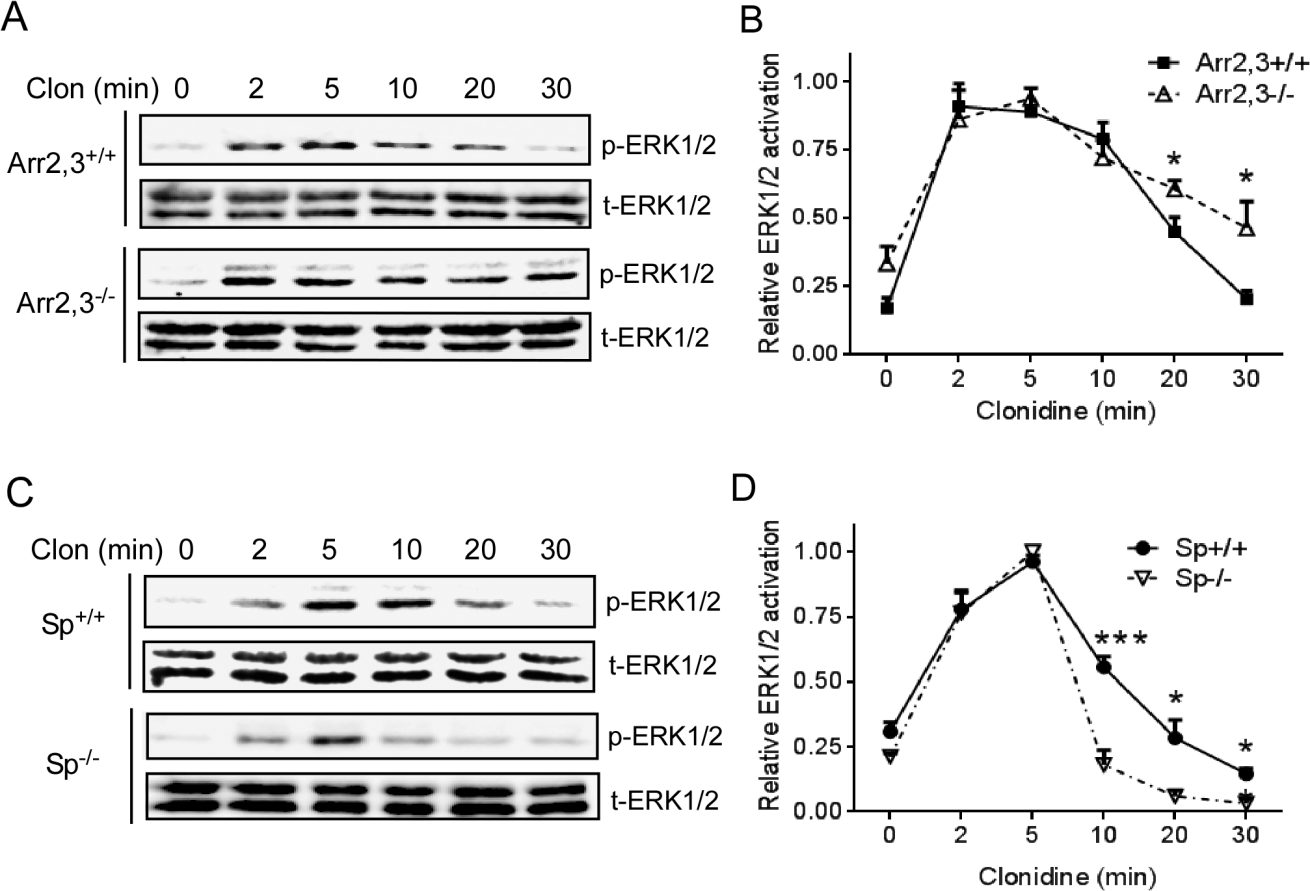
Spinophilin and arrestin reciprocally regulate α_2B_AR-induced ERK1/2 activation kinetics in MEFs. (A) Arr2,3^-/-^ and corresponding WT (Arr2,3^+/+^) MEFs expressing HA-α_2B_AR were stimulated with 1μM clonidine for indicated time points. Phospho- and total-ERK1/2 were detected by Western blots. Representative blots from multiple independent experiments are shown. (B) Quantitation of ERK1/2 activation in Arr2,3^+/+^ or Arr2,3^-/-^ MEFs at indicated time points. The relative ERK1/2 activation at each time point was expressed as a ratio to the peak level of ERK1/2 activation in the same experiment, which was arbitrarily defined as 1.0. Data were mean ± SEM. n = 4 for each condition. *, *p*<0.05, Arr2,3^-/-^ *vs*. Arr2,3^+/+^. (C) Sp^-/-^ and corresponding WT (Sp^+/+^) MEFs expressing HA-α_2B_AR were stimulated. Representative blots for phospho- and total ERK1/2 from multiple independent experiments are shown. (D) Quantitation of ERK1/2 activation in Sp^+/+^ or Sp^-/-^ MEFs at indicated time points. Data were mean ± SEM. n = 7 for data collected in Sp^+/+^ cells and n = 4 for Sp^-/-^ cells. *, *p*<0.05; ***, *p*<0.001, Sp^-/-^ *vs*. Sp^+/+^.

However, spinophilin may regulate α_2B_AR phosphorylation through mechanisms other than competing against arrestin, given that it contains multiple functional domains in addition to the receptor binding region [38, 39]. To address this, we examined whether the receptor binding region of spinophilin (Sp156-444) alone can sufficiently regulate α_2B_AR phosphorylation. In HEK293 cells overexpressing Myc-Sp156-444, the levels of α_2B_AR phosphorylation in response to agonist stimulation were significantly reduced compared to those in control cells expressing the empty vector (Fig 3A and 3B). This effect on α_2B_AR phosphorylation caused by the receptor binding region of spinophilin is comparable to that caused by the full length spinophilin (comparing Fig 2D and Fig 3B). Furthermore, in CosM6 cells (which express a low level of endogenous β-arrestins), overexpression of Myc-Sp156-444 failed to alter agonist-induced α_2B_AR phosphorylation (Fig 4C). This suggests that the inhibitory effect of the receptor binding domain of spinophilin on α_2B_AR phosphorylation requires a relatively high level of arrestin expression to be detected. Taken together, these data strongly support that spinophilin attenuates α_2B_AR phosphorylation through competition against β-arrestins in cells.

### Spinophilin counteracts arrestin-dependent desensitization of α_2B_AR-induced ERK1/2 activation

We next examined the effect of spinophilin and β-arrestins on α_2B_AR signaling. In MEFs expressing both spinophilin and arrestins, activation of the α_2B_AR by clonidine induced transient ERK1/2 activation, which was desensitized after 20 min of stimulation (Fig 4). In MEFs without β-arrestin expression (Arr2,3^-/-^), ERK1/2 signaling was prolonged after a 30-min treatment (Fig 4A and 4B) as compared to that in the corresponding Arr2,3^+/+^ MEFs, suggesting that β-arrestins are required for terminating α_2B_AR signaling. On the other hand, in cells without spinophilin expression (Sp^-/-^), α_2B_AR-induced ERK1/2 activation was quickly desensitized at the 10 min time point (Fig 4C and 4D). This desensitization of ERK1/2 signaling is much faster than that in the corresponding Sp^+/+^ MEFs, suggesting an opposing effect of spinophilin on arrestin-dependent desensitization. We obtained similar results with other α_2_ agonists, including epinephrine and UK14,304 (data not shown). The activation rate of α_2B_AR-induced ERK1/2 signaling seemed not altered in Arr2,3^-/-^ or Sp^-/-^ cells. This is different from what we have previously observed for the α_2A_AR-induced ERK1/2 activation, which was accelerated in Sp^-/-^ cells but slowed in Arr2,3^-/-^ cells [7]. Taken together, our data suggest that reciprocal regulation by spinophilin and β-arrestins can have differential impacts on signaling evoked by the closely related α_2_AR subtypes.

### The Del301-303 α_2B_AR exhibits preferential interaction with spinophilin over arrestin

**Fig 5:**
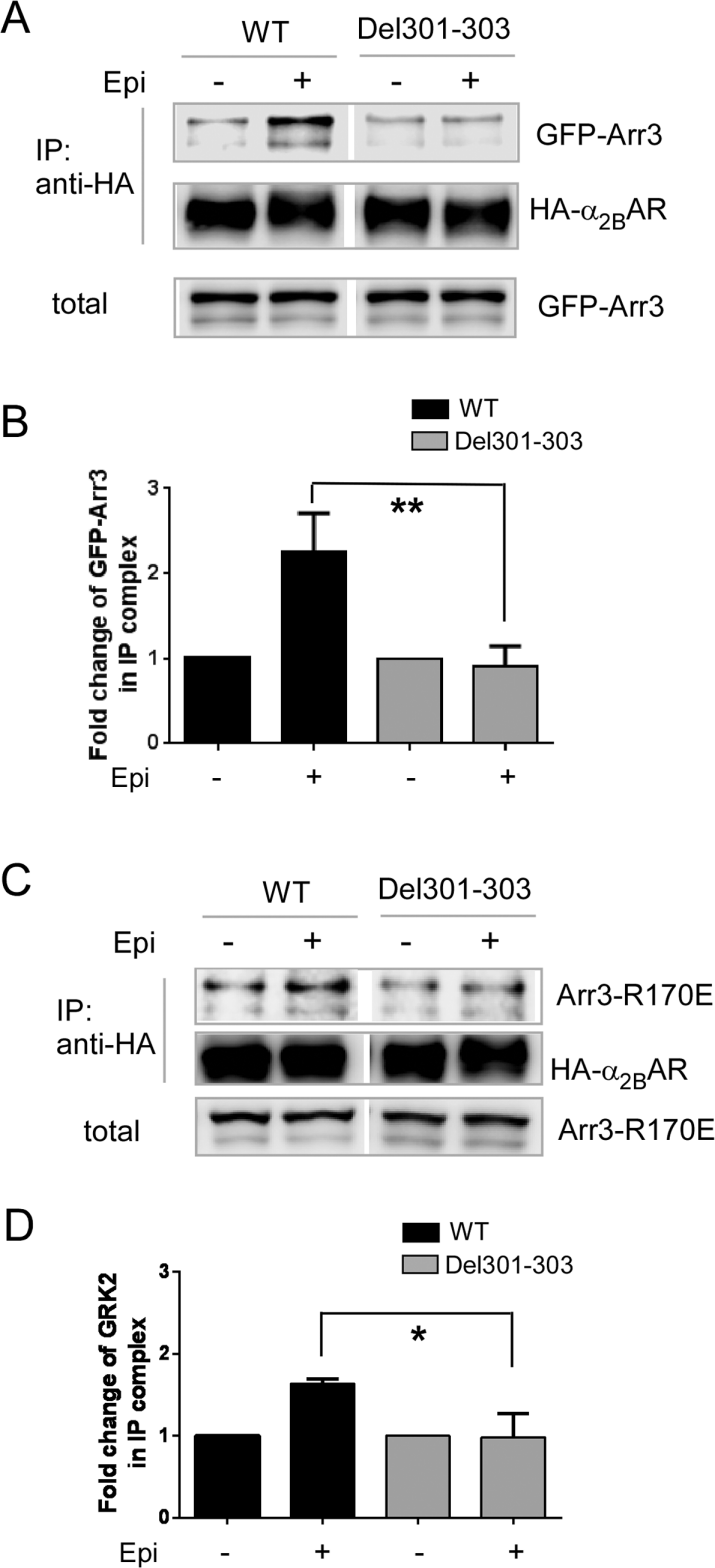
The Del301-303 α_2B_AR shows impaired interaction with arrestin 3. (A) Agonist treatment failed to promote interaction of the Del301-303 α_2B_AR with arrestin 3. Cells co-expressing GFP-tagged arrestin3 (GFP-Arr3) with HA-tagged WT α_2B_AR or Del301-303 α_2B_AR were stimulated with 100μM epinephrine (plus 1μM propranolol to block βARs), and the interaction between arrestin and either WT or Del301-303 α_2B_AR was examined by co-IP assays. (B) Quantitation of the agonist-induced fold change of GFP-Arr3 in the IP complex with the WT or Del301-303 α_2B_AR. n = 3–4 for each condition. **, *p<*0.01, WT *vs*. Del301-303. (C) Del 301–303 α_2B_AR was unable to interact with constitutively active mutant arrestin3 R170E following agonist stimulation. Cells co-expressing GFP-Arr3R170E together with WT or Del301-303 α_2B_AR were stimulated with 100μM epinephrine (plus 1μM propranolol). (D) Quantitation of the agonist-induced fold change of GFP-Arr3R170E in the IP complex with the WT or Del301-303 α_2B_AR. n = 3–4 for each condition. *, *p<*0.05, WT *vs*. Del301-303.

The human variation Del301-303 α_2B_AR displays reduced phosphorylation and desensitization following agonist treatment [27]. Given the importance of β-arrestins for α_2B_AR phosphorylation and desensitization demonstrated above, we predicted that this receptor would show impaired interaction with β-arrestins. Indeed, while epinephrine markedly enhanced the amount of arrestin 3 associated with the WT α_2B_AR, such treatment failed to increase the association of arrestin 3 with the Del301-303 α_2B_AR (Fig 5A and 5B). Impaired arrestin binding to the Del301-303 α_2B_AR may be a consequence of the decreased phosphorylation level of this receptor. Alternatively, Del301-303 may cause conformational changes that reduce its binding to arrestins independent of receptor phosphorylation. To address this possibility, we examined the ability of Del301-303 α_2B_AR to interact with the phosphorylation-insensitive arrestin 3 (Arr3R170E). Replacement of Arg170 with a Glu results in constitutive binding of arrestin 3 to agonist-activated GPCRs even in the absence of receptor phosphorylation [40, 41]. Epinephrine treatment significantly enhanced interaction of Arr3R170E with the WT α_2B_AR (Fig 5C and 5D). However, such treatment had no effect on Arr3R170E interaction with the Del301-303 α_2B_AR (Fig 5C and 5D). These data suggest that Del301-303 changes the conformation of the receptor leading to a diminished affinity for β-arrestin binding.

**Fig 6:**
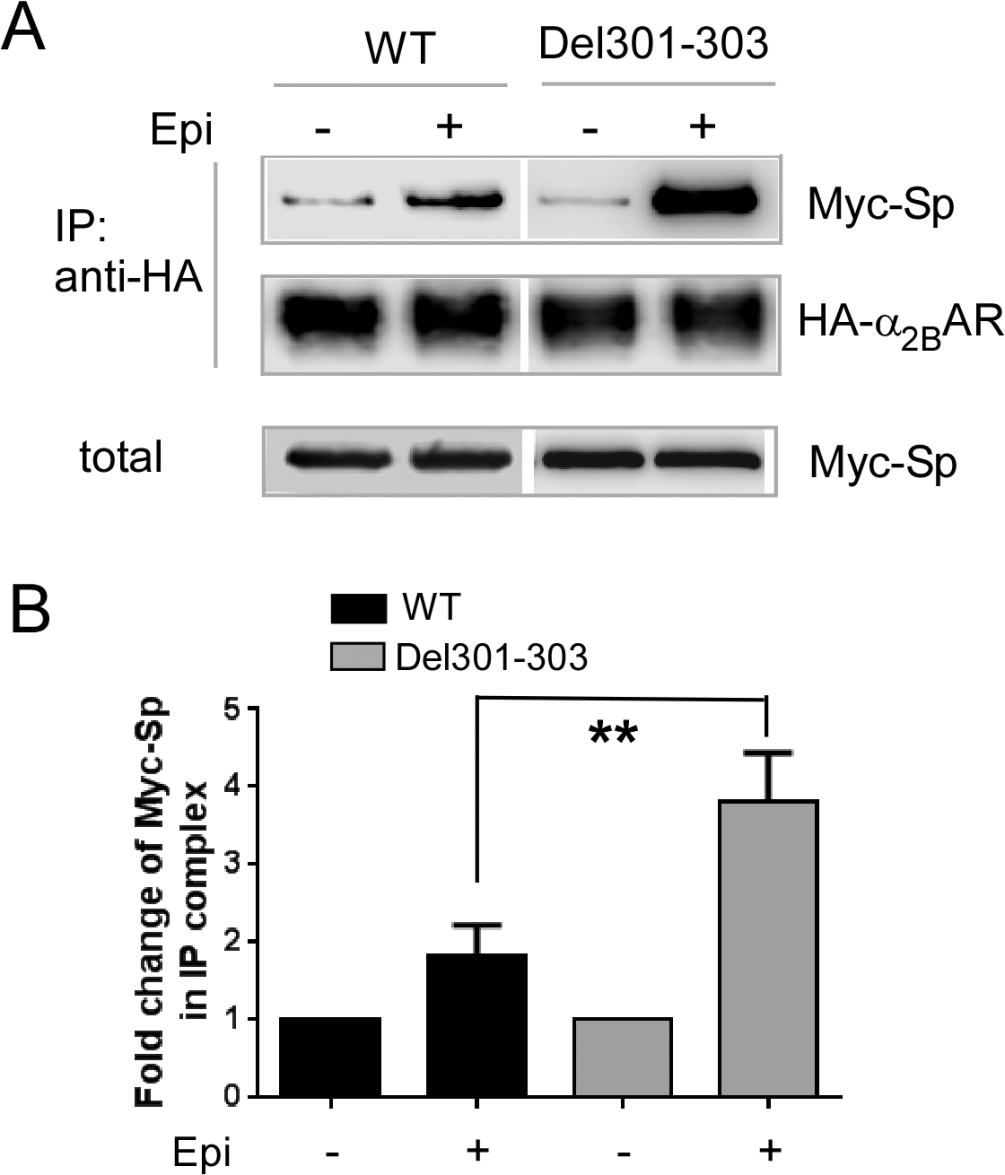
Del301-303 α_2B_AR has a stronger interaction with spinophilin than WT α_2B_AR. (A) Cells co-expressing Myc-spinophilin together with HA-tagged WT or Del301-303 α_2B_AR were stimulated with 100μM epinephrine (plus 1μM propranolol to block βARs). (B) Quantitation of the agonist-induced fold change of Myc-spinophilin in the IP complex with the WT α_2B_ or Del301-303 α_2B_AR. n = 3–5 for each condition. **, *p<*0.01, WT *vs*. Del301-303 α_2B_.

Based on the reciprocal effect of spinophilin and arrestin on receptor interaction, we predicted that the Del301-303 α_2B_AR would have a higher affinity for spinophilin binding. As expected, interaction of spinophilin with the Del301-303 α_2B_AR was significantly increased when compared to that with the WT α_2B_AR (Fig 6). Taken together, our data suggest biased interaction of the Del301-303 α_2B_AR with spinophilin.

### Spinophilin is essential for sustaining the prolonged ERK1/2 signaling elicited by the Del301-303 α_2B_AR

**Fig 7:**
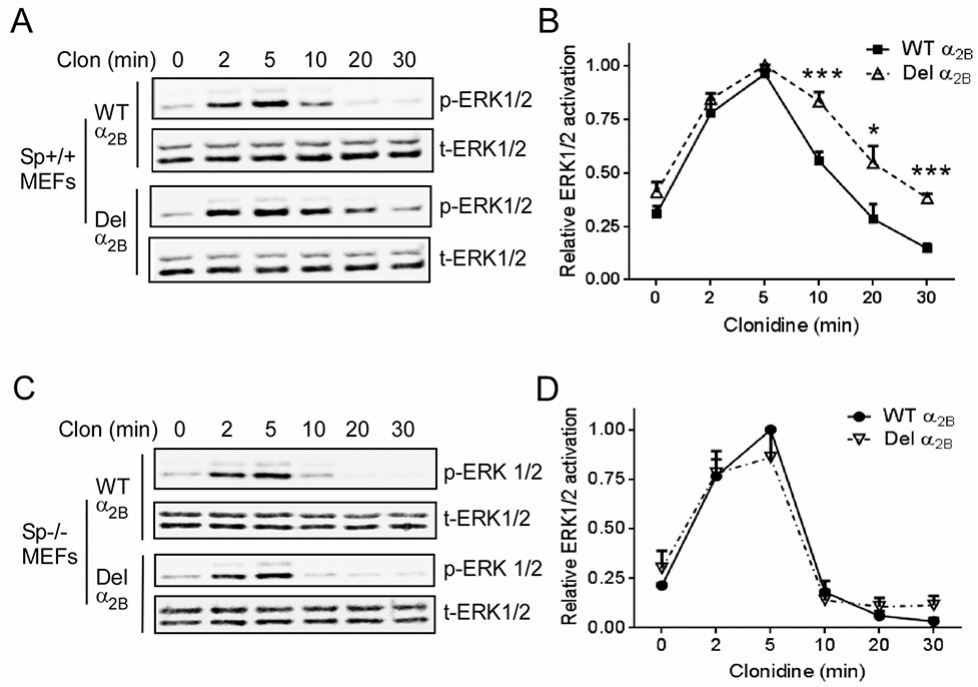
Spinophilin is required for maintaining the sustained ERK1/2 activation induced by the Del301-303 α_2B_AR in MEFs. (A) Sp^+/+^ MEFs expressing the WT or Del301-303 α_2B_AR were stimulated with 1μM clonidine for indicated time points. Representative blots show phospho- and total-ERK1/2. (B) Quantitation of ERK1/2 activation in Sp^+/+^ MEFs. The relative ERK1/2 activation at each time point was expressed as a ratio to the peak level of ERK1/2 activation in the same experiment, which was arbitrarily defined as 1.0. n = 7 for the WT α_2B_ group and n = 6 for the Del301-303 α_2B_ group. *, *p*<0.05; ***, *p*<0.001, WT α_2B_ *vs*. Del301-303 α_2B_. (C) Sp^-/-^ MEFs expressing the WT or Del301-303 α_2B_AR were stimulated with 1μM clonidine for indicated time points. Representative blots show phospho- and total-ERK1/2. (D) Quantitation of ERK1/2 activation in Sp^-/-^ MEFs. n = 4 for the WT α_2B_ group and n = 3 for the Del301-303 α_2B_ group.

The above data suggest that the diminished affinity of the Del301-303 α_2B_AR for β-arrestin binding likely underlies impaired desensitization of signaling elicited by this receptor variant. Since the Del301-303 α_2B_AR showed increased interaction with spinophilin, we further sought to address whether spinophilin binding to this receptor plays a role in sustaining its prolonged signaling. We therefore examined the kinetics of ERK1/2 signaling elicited by the WT or Del301-303 α_2B_AR in Sp^-/-^ and the corresponding Sp^+/+^ MEFs. Consistent with the reduced desensitization of the Del301-303 α_2B_AR signaling reported previously [27], ERK1/2 activation elicited by the Del301-303 α_2B_AR was prolonged when compared to that elicited by the WT α_2B_AR in Sp^+/+^ MEFs (Fig 7A and 7B). Strikingly, in Sp^-/-^ MEF, we failed to observe any difference in ERK1/2 activation kinetics induced by the Del301-303 versus the WT α_2B_AR (Fig 7C and 7D). In both cases, ERK1/2 signaling was quickly desensitized (Fig 7C and 7D). These data suggest that spinophilin is required for sustaining the prolonged ERK1/2 signaling elicited by the Del301-303 α_2B_AR.

### The α_2B_AR-elicited hypertensive response is enhanced in arrestin 3 null mice but diminished in spinophilin null mice

Given the reciprocal effects of spinophilin and β-arrestins in regulating α_2B_AR phosphorylation and desensitization, we sought to further determine the role of these proteins in regulating the α_2B_AR-elicted hypertensive response. We first compared the level of increase in blood pressure (a response known to be elicited by the α_2B_AR [18]) following administration of an α_2_ agonist, UK14,304, in Arr3^-/-^ and the corresponding WT (Arr3^+/+^) mice in the same genetic background. In Arr3^-/-^ mice, UK14,304 induced a significantly higher increase in the mean arterial pressure (MAP) compared to that in Arr3^+/+^ mice (Fig 7A and 7B). Furthermore, this α_2B_AR-elicited hypertensive response appeared to last for a longer time in Arr3^-/-^ mice compared to WT mice (Fig 7A), and the peak area under the hypertensive curve (AUC) was more than twice of that in WT mice (Fig 7C). These data suggest that the α_2B_AR-elicited hypertensive response is enhanced and prolonged in the absence of arrestin 3, supporting an *in vivo* role of arrestin 3 in desensitizing this effect.

**Fig 8:**
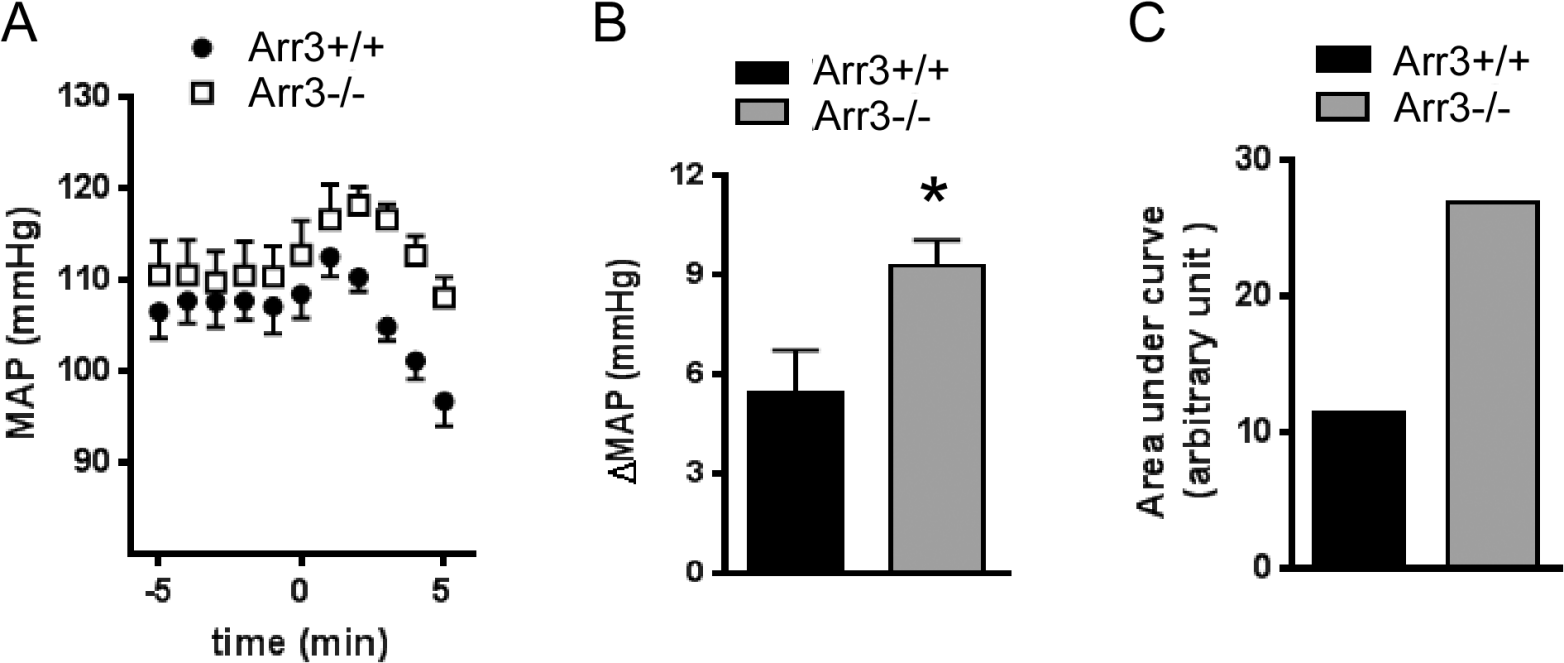
The α_2B_AR dependent hypertensive response is enhanced in arrestin 3 deficient mice. (A) Mean arterial pressure (MAP) measured in Arr3^-/-^and corresponding Arr3^+/+^ mice in the same genetic background after injection of UK14,304 (0.1mg/kg i.v.). (B) Quantitation of agonist-induced changes in MAP(ΔMAP) over the basal level. (C) Quantitation of area under curve of the hypertensive response curve. n = 5 for each group. *, *p* <0.05, Arr3^+/+^ *vs*. Arr3^-/-^.

We then compared the level of increase in blood pressure induced by UK14,304 in Sp^-/-^ and the corresponding WT (Sp^+/+^) mice in the same genetic background. UK14,304-induced change in MAP in Sp^+/+^ mice were somewhat higher than that in Arr3^+/+^ mice (7.4 ± 2.5 vs 5.4 ± 2.9, *p* = 0.28), likely due to the difference in genetic background between these lines. In Sp^-/-^ mice, the α_2B_AR-elicited hypertensive response was greatly diminished (Fig 8A and 8B) and the AUC in these mice was dramatically reduced as compared to those in Sp^+/+^ mice (Fig 8C). These data strongly suggest that the *in vivo* α_2B_AR responsiveness requires the presence of spinophilin; in mice without spinophilin expression, the α_2B_AR cannot elicit an effective hypertensive response to α_2_ ligands.

## Discussion

Using the α_2A_AR subtype as a model, we previously identified competition between spinophilin and β-arrestins for interaction with the 3i loop of the receptor [8]. In this study, we confirmed the mutually exclusive binding of these proteins to the 3i loop of the α_2B_AR subtype (Fig 1), and validated that β-arrestins promoted, whereas spinophilin attenuated, agonist-induced α_2B_AR phosphorylation (Fig 2). In addition, we demonstrated that the receptor-binding domain of spinophilin sufficiently attenuated α_2B_AR phosphorylation (Fig 3), which further supports the notion that spinophilin regulates receptor phosphorylation through competition against arrestin binding to the receptor.

Consistent with a role of β-arrestins in desensitizing receptor signaling, we found that α_2B_AR-mediated ERK1/2 activation was prolonged in cells without β-arrestins expression (Fig 4A and 4B). In cells without spinophilin expression, this signaling process was quickly desensitized (Fig 4C and 4D), presumably due to enhanced arrestin binding to the receptor in these cells. In our previous studies, activation of ERK1/2 signaling by the α_2A_ subtype was accelerated in spinophilin deficient cells but slowed in arrestin deficient cells [7]. However, we did not observe these changes for α_2B_AR-induced ERK1/2 activation, suggesting that the signaling profiles induced by closely related α_2_AR subtypes are differentially regulated by spinophilin and β-arrestins.

The α_2B_AR mediates the hypertensive response to α_2_ ligands [18]. The human Del301-303 α_2B_AR variant, which exhibits reduced phosphorylation and desensitization profiles *in vitro* [27] (also Fig 7A and 7B) and *in vivo* [42], has been associated with early onset hypertension in a Swedish population [23, 24]. We found that this variant showed impaired interaction with arrestin 3 (Fig 5A). In particular, the Del301-303 α_2B_AR failed to interact with the constitutive form of arrestin 3 that is insensitive to receptor phosphorylation (Fig 5B), suggesting that the conformational change caused by Del301-303 alters the intrinsic affinity of the receptor to arrestins. By contrast, the Del301-303 α_2B_AR showed a much stronger interaction with spinophilin (Fig 6). Strikingly, in cells without spinophilin expression, ERK1/2 activation induced by the Del301-303 α_2B_AR was quickly desensitized with a profile similar to that induced by the WT receptor (Fig 7C and 7D). These data suggest that spinophilin interaction is essential for maintaining the prolonged signaling profile induced by this receptor variant, and further indicate that spinophilin may represent an attractive target in manipulating functions of this polymorphic variant.

**Fig 9:**
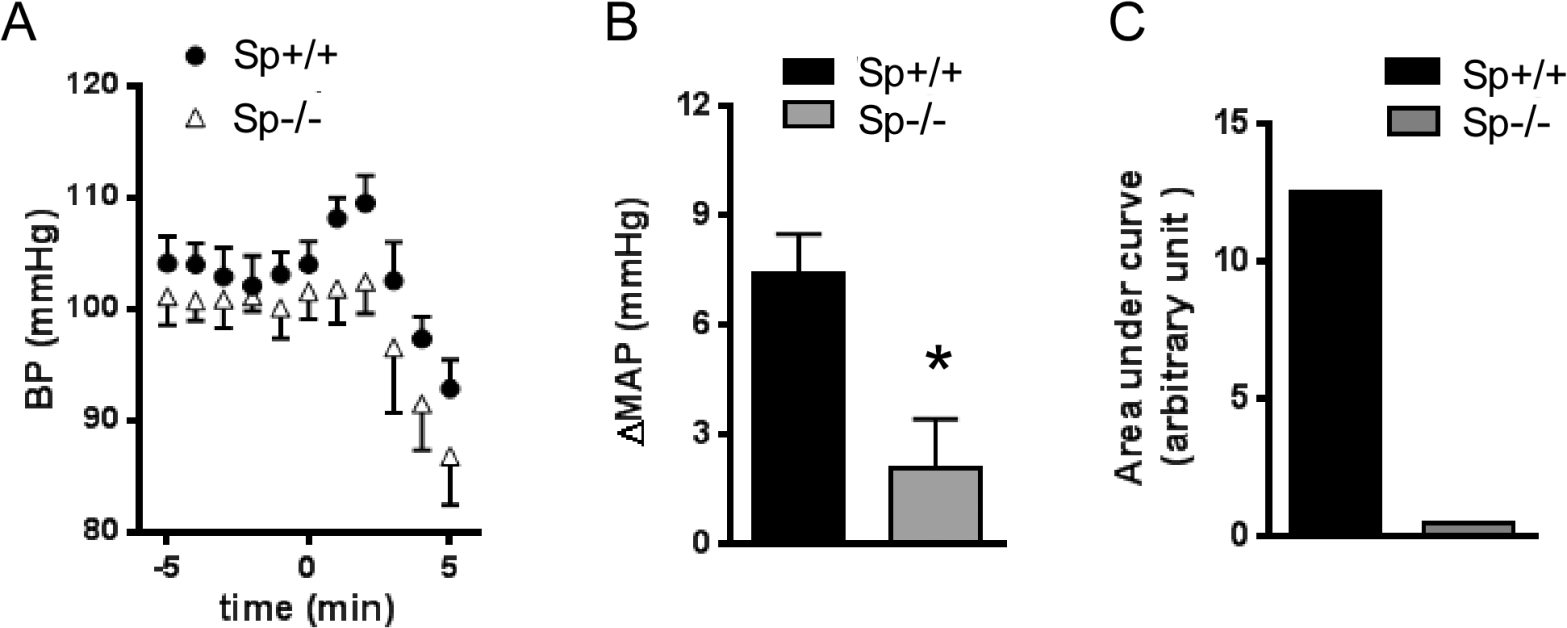
The α_2B_AR dependent hypertensive response is diminished in spinophilin deficient mice. (A) Mean arterial pressure (MAP) measured in Sp^+/+^ and Sp^-/-^ mice in the same genetic background after UK14,304 injection (0.1mg/kg i.v.). (B) Quantitation of agonist-induced changes in MAP(ΔMAP) over the basal level. (C) Quantitation of area under curve of the hypertensive response curve. n = 5 for each group. *, *p* <0.05, Sp^+/+^ *vs*. Sp^-/-^.

We previously found that multiple *in vivo* responses elicited by the α_2A_AR subtype are potentiated in spinophilin deficient mice, but dampened in arrestin 3 deficient mice where spinophilin binding to the receptor is enhanced [7, 9, 10]. Particularly, we have found that spinophilin attenuates the α_2A_AR-dependent hypotensive response; in spinophilin null mice, this response is highly potentiated [9]. This is the opposite of what we have observed for responses elicited by the α_2B_AR subtype in the current study. In spinophilin null mice, the α_2B_AR-elicited hypertensive response was nearly abolished (Fig 9), whereas in arrestin 3 deficient mice, this response was enhanced and prolonged (Fig 8). Our current data suggest that interaction with spinophilin is indispensable for α_2B_AR to elicit the hypertensive response. Collectively, our previous and current studies suggest that spinophilin regulation of the closely related α_2_AR subtypes can result in distinct functional outcomes *in vivo*. Diminished regulation by spinophilin enhances the hypotensive effect elicited by the α_2A_ subtype [9] while reducing the counteracting hypertensive effect by the α_2B_ subtype (Fig 9). Hence, reducing spinophilin binding to the α_2_AR subtypes may represent a useful therapeutic strategy for treatment of hypertension. This strategy may be particularly beneficial to the hypertensive population with the spinophilin-biased variation of α_2B_AR, Del301-303, given the essential role of spinophilin in sustaining signaling by this receptor variant (Fig 7C and 7D).

We tested multiple α_2_ ligands in this study. Although all these ligands promoted binding of both arrestin and spinophilin and gave similar results in our experimental readouts, it should be noted that these ligands likely exhibit different biases for the arrestin pathway, as described for the α_2C_AR subtype previously [43]. Further investigation is needed to quantitatively compare the functional selectivity of these ligands to the α_2B_AR, including arrestin and spinophilin recruitment. Our studies suggest that ligands that lead to stronger biased interaction of the α_2_AR subtypes with β-arrestins over spinophilin would be more beneficial for hypertension treatment than traditional ligands that can enhance binding of both proteins to the receptor. Identifying such ligands may represent a new direction of therapeutic development for treatment of hypertension.
